# Recommended content of referral letters from general practitioners to specialised mental health care: a qualitative multi-perspective study

**DOI:** 10.1186/1472-6963-13-329

**Published:** 2013-08-19

**Authors:** Miriam Hartveit, Olav Thorsen, Eva Biringer, Kris Vanhaecht, Benedicte Carlsen, Aslak Aslaksen

**Affiliations:** 1 Research Network on Integrated Health Care in Western Norway, Helse Fonna HF, Valen Hospital, N-5451, Haugesund, Valen, Norway; 2 Department of Public Health and Primary Health Care, Faculty of Medicine and Dentistry, University of Bergen, Bergen, Norway; 3 Section of Mental Health Research, Helse Fonna HF, Haugesund, Norway; 4 School of Public Health, KU Leuven, University of Leuven, Leuven, Belgium; 5 Uni Rokkan Centre, Bergen, Norway; 6 Department of Radiology, Haukeland University Hospital, Bergen, Norway; 7 Institute of Surgical Sciences, Faculty of Medicine and Dentistry, University of Bergen, Bergen, Norway

**Keywords:** Referral and consultation, Mental health, Health services, General practice, Group interview

## Abstract

**Background:**

In most Western countries, the referral letter forms the basis for establishing the priority of patients for specialised health care and for the coordination of care between the services. To be able to define the quality of referral letters, the potential impact of the quality on the organisation of care, and to improve the quality of the letters, we need a multidimensional definition of the ideal content. The study’s aim was to explore what information is seen as most important and should be included in referral letters from primary care to specialised mental health care to facilitate prioritisation and planning of treatment and follow-up of the patients.

**Methods:**

Based on purposive sampling, four mixed discussion groups, which included general practitioners, mental health nurses from primary health care, psychiatrists and psychologists from specialised mental health care, managers and patient representatives, were formed; they were asked to identify the information they considered important in a mental health referral letter. In line with the Delphi technique, the importance of the themes was later individually rated by the participants. The study was conducted within The Western Norway Regional Health Authority.

**Results:**

The four groups identified 174 information themes. After excluding themes that were assessed as duplicates, replaceable or less important, 40 themes were suggested, organised in seven units. A set of check-off points of essential information is recommended as an introduction in the referral letter.

**Conclusion:**

Compared with general guidelines and guidelines for somatic care, the results of this study suggest that the referral letter to specialised mental health care should have a larger emphasis on the overall treatment plan, on the specific role of specialised health care in the continuum of care, and on patient involvement. Further research should evaluate the validity of these findings for other patient groups in need of integrated care and investigate how the quality of referral letters affects patient-related and organisational outcomes.

**Trial Registration:**

Trial Registration number: NCT01374035

## Background

Patients suffering from mental disorders are one of the largest patient groups worldwide and constitute a significant contribution to the global burden of disease [1]. Provision of equal healthcare to those with equal needs (horizontal equity) and sufficient accessibility to specialised mental health care is therefore important not only to the individual patients and their relatives, but also to the society [1,2]. Within a health care system where specialist health care is a limited recourse, it is of great importance that the patients most in need are prioritised. In Norway, as in many Western countries, the prioritisation is conducted in two steps: first, a General Practitioner (GP) decides if a patient should be referred; and second, a specialist decides if and when the patient should receive specialist health care. For both steps, the GP needs to know what information the referral letter should include [3]. However, studies on the content of referral letters suggest that they lack important information [4-6]. The consequences of low quality referral letters are to a large degree unknown, but a positive correlation has been found between the content of referral letters and the specialists’ confidence that they have enough information to make the correct priority for patients receiving cancer care [7]. A recent study by Holman et al. revealed a fair inter-rater reliability between specialists prioritisation of patients based on referral letters within mental health care [8], indicating a risk of low horizontal equity. Both studies suggest defining guidelines for the content of referral letters as one strategy to improve the process of prioritisation [7,8].

During recent decades, suggestions as to what information referral letters ideally should include have been put forward, but variable quality of referral letters seems to be persistent [5,6]. According to Øvretveit’s definition, quality in healthcare involves three perspectives: professional, patient, and management [9]. Guidelines, for instance for the ideal content of referral letters, should be defined by consensus of representative health professionals, patients, and managers. Studies revealing a discrepancy within the professional perspective regarding the appropriate content of referral letters indicate that both GPs and hospital specialists should be represented [10-12]. However, existing studies on the quality of referral letters are often based on a standard determined and defined by health personnel alone or only by hospital specialists [4,6]. Another barrier for improving the content of referral letters could be the extent and numbers of different guidelines that GPs are expected to comply with, if all specialities define their own local guidelines. By contrast, using scientific methods to define one standard of the most important information by consensus between the involved parties is in accordance with effective quality improvement [9,13,14].

By legislation, the prioritisation of patients in Norway is based on an assessment of (i) the condition and its influence on quality of life, (ii) the expected effect of recommended interventions (utility), and (iii) the cost-effectiveness of suggested intervention [15,16]. “The good referral letter” is a Norwegian recommended guideline for the content of referral letters [17]. It is a general form for both somatic and psychiatric care, and includes the patient’s personal information, information on the referring doctor, special information (allergy), diagnosis, expected treatment, relevant information on the patient’s situation and condition, the level of pain or problems, and the degree of urgency [17]. It does not focus on patient experiences as suggested in mental health referral letters [18].

To be able to explore the consequences of the quality of referral letters and to improve the quality of referral letters, we need a valid definition of the most important information it must include [13,19]. The study is an example of a procedure for the first two steps in quality improvement: select the target area and define recommended practice (see Table 1 “The first two steps in quality improvement”). The aim of the present study was to identify what a referral letter to specialised mental health care should include to provide the necessary information to correctly and sufficiently prioritise and plan treatment and follow-up of patients, as perceived by patients, health professionals and managers.

**Table 1:** The first two steps in quality improvement

**Theory based steps**	**In this case**
**1. Select target area**	
Frequency	Large patient population [1], referral letters used whenever need for specialised health care.
Importance	Large suffering, large impact on society [1]
Complexity	Many stakeholders, inter-organisational
Insufficiently effective, efficient, accessible, acceptable/patient-centred, equitable, and/or safe care [28]	Risk of incorrect prioritisation of patients and inappropriate care [7,8]
Expected improvement potential [13]	Studies reveal that referral letters in general and within mental health care lack important information [4-6]
**2. Determine/define recommended practice**	
Explore existing knowledge [13]	Literature review revealed no evidence-based recommended standard for content of referral letters to specialised mental health care
If not sufficient knowledge: Define recommended practice	Structured group interview using the method Language Processing [20]
A. Involve valid perspectives [9]:	Including:
Professional	Health professionals from primary care (GPs and mental health nurses) and from specialist mental health care (psychiatrist and trained psychologists)
Patient/client	Patient representatives from Mental Health Patient Organisation
Organisational	Operating managers within specialist mental health care.
B. Make feasible [13]	Delphi process to determine the most important content. Exclusion of themes where less than 75% of participants have rated them as highly important [22].

The theoretical framework for the method employed in this study.

## Methods

The study consisted of two steps. First, structured group interviews [20] with mixed groups representing patients, health professionals and managers were conducted. Second, the Delphi method (a postal questionnaire method where suggested items are reviewed by the same participants for a second rating) [20] was used to prioritise the suggested themes from the group interviews. The study was conducted within the Western Norway Regional Health Authority (population: 1 million). We used purposive sampling [20] based on Øvretveit’s defined perspectives of quality in health care [9]. Health professionals from both primary health care (GPs and mental health nurses) and specialised mental health care (psychiatrists and trained psychologists) represented the professional perspective. Participants were enrolled by persons or organisations external to the study based on their experience and interest in the subject. Patient representatives and GPs were enrolled by their local organisations, while managers, mental health nurses in primary care and specialists were suggested by their managers. Participation was based on written informed consent according to the Helsinki Declaration [21]. The study was presented and accepted by the Norwegian Social Science Data Service and the National Committee for Medical and Health Research Ethics.

### Step 1: group interviews

The group interviews were conducted as consensus development panels, as defined by Bowling [20]. Four groups were formed and interviewed once in a nearby health care centre. There were 19 participants, whereas twelve were men. Nine of the participants were health professionals within primary or specialized mental health care, four patient representatives and six were managers. In addition to their professional titles, many had experience in several areas, e.g. managers or patient representatives that also have a health professional background. They were all experienced; almost half had more than 15 years of experience in their present position. The timeframe for an interview was predefined to last two to three hours. The interviews started with a short presentation of the participants, the study, and the purpose of referral letters. By brainstorming, participants wrote ideas on post-it notes to the question “What information do you think is important that the specialised mental health care receive in a referral letter?”. The ideas (information themes) were collected and reviewed in the group to ensure a common understanding of each theme. The participants were then encouraged to take part in open discussion on issues relevant to good referral letters and were given the opportunity to write down additional themes. At the end, the groups analysed their themes by grouping them and creating a heading for each group of themes. The interview was led by a senior mental health researcher (E.B.); while the categorising of themes was led by the group itself and was observed by a researcher (M.H.). One week after the second interview, the participants in the first two groups were phoned by a researcher and asked if they had additional themes. This part of the process did not result in any new themes and was discontinued. The input from the four groups was combined and analysed together by two researchers (M.H. and O.T.). First, inter-group duplicates were removed. Then, themes with equivalent meanings were made into single themes. Finally, by consensus, new common headings were developed based on the groups’ headings with the associated themes by two internal researchers (A.A. and M.H.) and one external researcher (O.H.). These headings were reviewed and approved by the researcher leading the group interviews (E.B.).

### Step 2: individual rating

Within the Delphi technique, the suggested information requirements that had been analysed and organised into the new headings and themes were sent to the participants [20]. They were asked to individually rate each theme’s importance on a scale from 0 (= “not important/irrelevant”) to 5 (= “extremely important/cardinal”). With the occurrence of a perceived overlap of themes, the participants were to place a “0” by the redundant theme and mark it with the number of the theme that should replace it. We started the analysis of the individual ratings by excluding the theme that the most participants considered replaceable and marking the theme that replaced it. We then excluded the theme that was seen as second most replaceable by the participants, and so on. Themes that were marked as “replacers” were not excluded. Those assessed as replaceable by only one or two participants were not excluded. Finally, we used a predefined cut-off limit of 75% or more of the participants scoring the themes at 4 or 5, as did Deneckere and colleagues [22]. Only the themes rated as most important or second most important by 75% or more of the participants were included.

## Results

**Figure 1:**
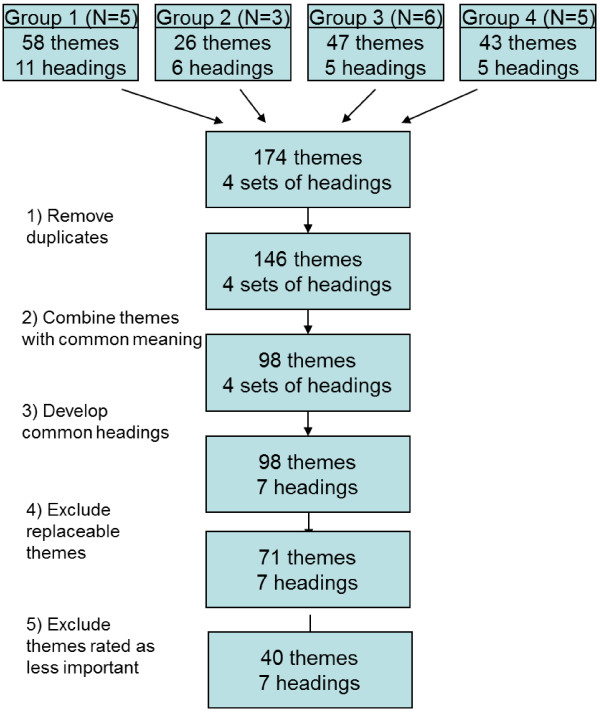
The reduction process.

Four group interviews were conducted with a total of 19 participants Seventeen completed the individual ratings using the questionnaire. The four groups suggested 174 themes. After excluding inter group duplicates and themes assessed as replaceable, 71 themes were left. Once we excluded themes that less than 75% of the respondents had rated as important (4 or 5 on the scale), we had a list of 40 themes that the participants considered as the most important information in a referral letter to specialised mental health care. The process is illustrated in Figure 1 “The reduction process”. The groups suggested from five to eleven headings. The four sets of headings with the suggested themes were analysed by three individual researchers. By consensus, seven headings were found to adequately cover the four sets:

•➢ Personal information and contact information.

•➢ Important introductory information (check-off points).

•➢ Case history and social situation.

•➢ Present state and results.

•➢ Past and on-going treatment efforts, involved professional network.

•➢ The patient’s assessment.

•➢ Reason for the referral.

**Figure 2:**
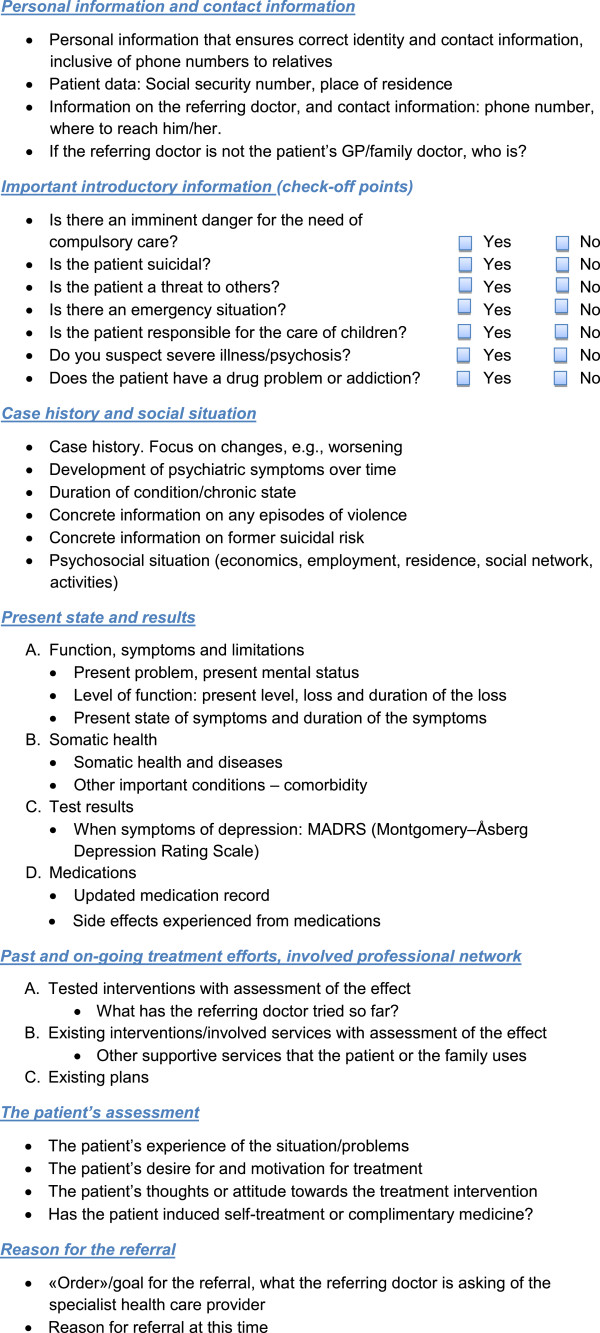
Suggested content of referral letters to specialised mental health care.

The main findings are that referral letters to specialised mental health care should include the overall plan for care, the involved services and interventions, and the patient’s preferences and goals as well as the regular information usually found in referral letters. An introductory section to the referral letter with check-off points about essential information was also suggested. The recommended information for a referral letter to specialised mental health care for adults, including the check-off points, is shown in Figure 2 “Suggested content of referral letters to Specialised Mental Health Care”.

## Discussion

The findings of the study identify referral letter information seen as important when referring a patient to specialised mental health care. The recommendations from the multi-perspective groups suggests a stronger emphasis on information about the planned integrated care, the specialist health care provider’s role in it, and on the patient’s involvement compared with “standard” referral letter forms. An introduction with seven check-off points on essential information also evolved during this study.

In contrast to many other efforts to define a standard for content of referral letters, we chose to invite the patients, the management and the professionals to give their perspective into the referral letter evaluation process. Since the referral letter is a communication means mainly between GPs and specialists, it can be argued that doctors are the only ones who are able to discern the type of information it should contain. However, there is increased focus on the importance of the patient perspective to aid in understanding and improving the quality of health care [9,23]. Another important aspect is health care management including knowledge about economic and legal opportunities and boundaries. Studies showing disagreement on appropriateness of referrals and the content of referral letters between stakeholders in the referral process support a comprehensive sampling [11,12]. We argue that the inclusion of all three perspectives gives a fuller description of the health care process and as such increases the validity of the results.

The groups were mixed to increase the richness of the data. Yet, there is the risk that the asymmetrical distribution of power that can be found within health care services could be maintained in the groups. To offset this possible asymmetry, and based on advice from patient representatives, we aimed at including at least two patient representatives in each group, but because of mitigating circumstances, achieved this in only two of the four groups. However, the large amount of input from the patient representatives gives us reason to believe that the effort to create a balance of power in the groups was successful. The definition of quality in health care, which forms the basis for the sampling method, is valid for all health care and therefore strengthens the generalisability of the findings. However, legislation, tradition and culture can affect which information is seen as important in a referral letter. The participants in this study were selected for their extensive experience and interest in the subject, and though this gave indepth insight into the topic, it can also at times be a barrier to innovative ideas and criticism within the topic. Another limitation in the study can be the type of services and professions represented in the sample. Other services such as unemployment agencies or social services may have information that could alter the results regarding assessment of important referral letter content. We argue that the results of this study are generalisable to health care systems similar to that of Norway. The method that we used to define the recommended content of referral letters, however, we argue is generalisable to health care in general.

There are many similarities between our findings, the Norwegian guideline “The good referral letter” [17], and international suggested standards within somatic care [5,24]. However, our study suggests more emphasis on the professional network, in which specialised health care is only one element. For instance, our informants thought information about services and interventions that the patient receives and about the overall care plan are important health care elements to convey when referring a patient to specialised mental health care. “The good referral letter” and other existing referral letter forms within somatic care do lend weak support also for our results concerning patient involvement [4,24], though some include information on the patient’s experience with the disease [6]. In contrast, the study on referral letters within mental health by Shaw and colleagues supports our patient focus findings [18]. The specific need for information regarding integrated care and patient involvement for patients with mental disorders highlights the need for a separate guideline for the content of referral letters within mental health care. However, it is noteworthy that similar information requirements have been found in other groups of patients who require shared or integrated care, such as the elderly [4]. Thorsen and colleagues defined three types of referral letters: a request for a specific assessment or treatment, an invitation to have a second opinion, and a request for mutual responsibility for the care of a patient [25]. Care for people that are referred to specialist mental health care usually requires cooperation between this service and primary health care [2]. Referral letters for these patients are therefore often a request for mutual responsibility for a period of time. Further research should explore if our results are valid for patients that are in need of integrated or shared care regardless of the diagnosis.

Optimal prioritisation of patients to ensure sufficient accessibility to specialised mental health care is important to patient safety. However, the structures and processes involved to support this are complex [26]. The present study focuses on one part expected to be relevant; the need for sufficient information to prioritise among patients. It suggests that there is some information seen as important when referring a patient to mental health care not emphasised in a general referral letter form. However, factors other than the content of referral letters can affect the setting of priorities, such as acquaintance with the referring GP [27]. Recognising that there may be many factors affecting the accessibility of specialised mental health care, the present study should be seen as one step in exploring best practices for one of the factors. Future research should emphasise exploration of the referral process in the following areas: (a) if or to what degree the content of referral letters have an impact on the quality of care [28] for patients with mental diseases; (b) the generalisability of our findings to other patient groups and context; and (c) if or to what degree other factors than the content of referral letters are relevant for the outcome of the referral process and the subsequent care for the patient.

## Conclusion

In this study, we have explored the information that a referral letter to specialised mental health should include. We have revealed some important elements of patient information and care that are not aspects of general referral letter forms. Our findings recommend a stronger focus on the on-going and planned care, so that specialised mental health care has a greater understanding of its role, and more emphasis on the patient’s assessment and preferences. Beyond that, a general form, like “The good referral letter”, can serve as a guideline. The recommended set of introductory check-off points can serve as a checklist for GPs when writing a referral letter and can outline essential information for specialists. However, it is important to evaluate if or to what degree high quality referral letters, according to the results of this study, improve the outcomes for patients and the organisation, and if they have a positive impact on the accessibility of specialised health care for the patients most in need.

## Competing interest

No competing interests are declared for any of the authors.

## Authors’ contributions

MH defined the protocol for the study, observed during the interviews, participated in the analysis of the data and drafted the manuscript. OT took part in the analysis. EB conducted the interviews, while KV made contributions to the development of the protocol together with BC. AA supervised and made important contributions during the study process. All authors have contributed during the writing and publishing process, and have approved the final manuscript.

## Pre-publication history

The pre-publication history for this paper can be accessed here:

http://www.biomedcentral.com/1472-6963/13/329/prepub
